# Self-Determined Health App Evaluation Questionnaire Development: Mixed Methods Study

**DOI:** 10.2196/63739

**Published:** 2025-07-01

**Authors:** Angelika Rzepka, Kurt Edegger, Stefan Welte, Diotima Bertel, Anja Mandl, Günter Schreier

**Affiliations:** 1 Center for Health & Bioresources, AIT Austrian Institute of Technology GmbH, Giefunggasse 4, Wien, 1210, Austria, 43 6648561598; 2 Center for Technology Experience, AIT Austrian Institute of Technology GmbH, Wien, Austria; 3 Gesundheitsfonds Steiermark, Graz, Austria

**Keywords:** health apps, app quality, DiGA, nutrition, sports, Mental Health, Symptom Checker, health applications, diet, symptom, questionnaire, evaluation, development, physical exercise, exercise, survey, quality assurance, user-friendliness

## Abstract

**Background:**

The rapid increase in the number of health apps and their volatility in Austrian pp stores for Android and Apple are signs of a flourishing business sector in the wellness industry.

**Objective:**

In this report, a questionnaire for informed decision-making by users was developed and evaluated using health apps in the categories “Nutrition”, “Exercise”, “Mental Health” and “Symptom Checker”.

**Methods:**

Evaluation criteria were derived from multiple reference documents and weighted in a survey, as well as a focus group meeting. Further, the selected evaluation criteria were tested against selected apps, which were most popular in the above-mentioned categories in fall 2023.

**Results:**

The short questionnaire is to be made publicly available to citizens and covers the categories of the quality of the app provider (regulatory compliance, safety, and quality assurance), the content quality of the app (functionality and evidence-based content), and the user-friendliness of the app. It consists of 6 question items, which appeared to be problematic in the evaluation most often. For ease of use, a short questionnaire with the most critical questions and helpful tips from the evaluation was expanded by the question of importance and with meaningful advice on how to find the right information in an app.

**Conclusions:**

The evaluation based on the comprehensive questionnaire using selected apps confirmed the importance of such a questionnaire since most of the apps lacked basic properties in terms of safety and security.

## Introduction

### Background

The global as well as national supply of health apps is overwhelming, and more and more people are using health apps. This raises the basic questions of which apps can be considered health apps, and how can the credibility of these apps be better assessed.

Fundamentally, a distinction is made between [1] health and mobile apps. Health apps are applications targeting consumers. They are intended to be used for preventive purposes and help promote or change lifestyle in a health-promoting manner.

On the other hand, medical apps are applications used to support “self-empowerment” (self-determination) and, for example, are supportive in coping with chronic diseases or in rehabilitation [2], which are subject to regulations like the medical device regulations.

The use of evaluation criteria for health apps is important for several reasons, as described below.

### Functionality

Evaluation criteria can assess the functionality of health apps to ensure that the intended tasks are effectively fulfilled. This could include monitoring health parameters, reminding users to take medication or providing personalized health recommendations.

### Quality assurance

Evaluation criteria help assess the quality of health apps. This is particularly important because the information and functions provided by health apps can have a direct impact on the health of users. Criteria enable ensuring that the apps provide accurate, reliable, and evidence-based information.

### Evidence-based content

Health apps should be based on scientific knowledge to provide accurate and reliable information. Evaluation criteria can verify whether the content presented in the apps is based on current scientific evidence.

### Usability and user experience

The usability of health apps is crucial for their effectiveness. Evaluation criteria can assess usability and accessibility to ensure that the apps are easily understandable and accessible. Furthermore, accessibility criteria may be important for allowing the apps to be used by impaired users.

### Privacy

Apps may process personal health data, so it is crucial to ensure that only the minimal set of data is acquired (“data minimization”) and that data are secured and protected in transit and at rest. Evaluation criteria can consider aspects such as data privacy, encryption, and compliance with regulatory requirements.

### Regulatory compliance

Apps, especially health apps may need to comply with certain legal and regulatory requirements. Evaluation criteria can assess whether the apps comply with applicable regulations and standards.

### Study Objectives

By applying evaluation criteria, users, health care providers, and regulatory authorities can make more- or better-informed decisions about the selection and use of health apps to ensure that they make a positive contribution to the health and well-being of users. Payors also need clear and transparent criteria to decide on reimbursement aspects.

The objective of this study was to establish evaluation criteria for users to make well-informed decisions in regard to health apps. Health app evaluation frameworks, such as the Mobile App Rating Scale (MARS) [3] and the ENLIGHT Tool [4], provide structured criteria for assessing functionality, usability, and information quality, typically for professional or research use; however, this work focuses on evaluation by laypersons rather than professional organizations, emphasizing user-friendly criteria for informed decision-making.

## Methods

### Ethical Considerations

Formal ethics approval was not required for this study because no patients or other vulnerable populations were involved. The user survey was completed anonymously by registered participants who volunteered to take part. No identifying information was collected at any stage, and all respondents provided informed consent before proceeding.

### Literature Search and Selection of Relevant Documents

The selection of evaluation criteria was conducted as part of a literature search based on the keywords “Bewertungskriterien Apps Gesundheit” (evaluation criteria health apps) and “evaluation criteria health Apps” in English. The search was carried out on the portals Google Scholar and PubMed, and additional literature references from publications were followed up based on the results.

The World Health Organization (WHO) includes “health apps” in the “Classification of Digital Health Interventions v1.0 (DHI)” [5,6]. This primarily categorizes apps according to target groups (eg, for clients or citizens or health care providers) and health system challenges, which then result in system categories for digital health applications.

According to the health system challenges level of health apps, “Lost client data” entails the interventions “1.1.3. Provision of individualized reminder services for clients” and “2.2.1 Longitudinal recording of health status and services of clients”, corresponding on a system level to the functions “Client Information System” and “Electronic Health Record”. The taxonomy used was based on [7] and the “Guidelines for reporting of health interventions using mobile phones: mobile health evidence reporting and assessment (mERA) checklist” [8].

These two documents generally refer to comprehensive mobile health (mHealth) applications with a strong focus on already manifested diseases.

Partially, reliance was placed on systematic reviews already conducted, such as health technology assessment frameworks for eHealth: a systematic review [9] or framework for supporting reimbursement decisions for digital health applications (mHealth) and its (retrospective) application to selected examples [1].

However, all of these encompass digital health applications, not just health apps. The existing assessment frameworks use different formats (checklists, questionnaires, and tables) and show differences in terms of the spectrum of evaluation domains, additional technology-specific aspects, and target groups. None of the frameworks are fully established or field-tested.

At the European level, there is also the initiative Label2Enable [10]. Label2Enable is an EU-funded project. Its task is to introduce CEN-ISO/TS 82304‐2 and its quality seal for health and wellness apps. The goal is to create a digital single market where high-quality health apps can be extensively used in prevention, health care, and self-care.

The ISO/TS 82304-2:2021(en) “Health software — Part 2 [11]: Health and wellness Apps—Quality and reliability” served as the basis for developing the evaluation criteria.

The following publications also served as the basis for the evaluation criteria:

1. Health apps. Basic paper with special consideration of the aspect of health literacy [12],
2. Opportunities and risks of health apps (CHARISMHA); German: chances and risks of mobile health apps (CHARISMHA) [13],
3. Deutsches Netzwerk Versorgungsforschung e. V. Memorandum - health and medical apps [2],
4. Health apps: How can certification look concretely: Learning from experiences here and elsewhere [14],
5. Digitization and patient safety - HE 2) Checklist for the use of health apps [15],
6. Evaluating, but how? - Criteria for the evaluation and reporting of studies on health and medical apps [16],
7. How good are health apps? What determines quality & risk? What guidance is available? [17],
8. Mobile app rating scale: a new tool for assessing the quality of health mobile apps [18],
9. Methods box health literacy [19],
10. Health apps: significant lever for patient empowerment - potentials, but hardly used so far [20],
11. Professional checklist good health information [21].

### Development of Evaluation Criteria

From the above publications, the evaluation criteria mentioned therein were entered into an Microsoft Excel file and assigned to overarching categories. In the next step, the discovered evaluation criteria were harmonized and evaluated. The evaluation criteria with the most matches were included in the list of evaluation criteria and categorized again. The preliminary evaluation criteria were presented and discussed in a focus group, consisting of the project team, health information experts, and public health experts. The evaluation criteria agreed upon in the focus group were prioritized within the focus group meeting and further refined in a quantitative survey of a total of 54 experts with different professions to filter out criteria considered less relevant and to make a final selection. Based on the results of the focus group and the survey, the evaluation criteria were revised and finalized and ended up in a long questionnaire, which is described in the results section.

### Evaluation of theQuestionnaire With Selected Apps

The developed questionnaire was evaluated with apps from the following categories: (1) Nutrition, (2) Exercise, (3) Mental Health, and (4) Symptom Checker. These categories were chosen because they cover the main fields of healthy lifestyle. The selection of apps aimed to evaluate the most used apps in Austria in these categories. An attempt to select apps based on existing databases was unsuccessful because the data were at least one year old (eg, Statista [22]) or the databases used a distribution model that was not applicable in this context (eg, data.ai [23]).

For these reasons, an exploratory approach was chosen where 11 individuals from the project team, selected to ensure as much diversity as possible in terms of mobile phones, operating systems, and histories of installed apps, participated. This group consisted of 6 males and 5 females, with 6 participants using the Google Play Store and 5 using the Apple App Store. Each individual searched for the respective keywords in their personal app stores, ensuring a broad variety of search habits and preferences, and recorded the results—15 apps per category—in an Excel spreadsheet. The results were weighted using a pivot table after data cleaning (typos, adding app manufacturers). Then, it was ensured that the top 10 apps in each category were available in both app stores and could be used for evaluation.

The following inclusion and exclusion criteria were additionally considered in the app selection, as listed in Textbox 1:

Textbox 1. **Inclusion and exclusion criteria for app selection**

**Inclusion criteria**

- The app must target humans as its audience (excluding apps for pets or similar).
- The app must be available in English or German.
- The app must be available in Austria in the Apple App Store and Google Play Store.

**Exclusion criteria**

- The app must not require additional hardware (eg, fitness watches, blood glucose meters, etc).
- The app must not be explicitly used for product sales.
- The purpose of the apps must not be to assist with insurance processes (ie, claim management).
- The apps must not require a diagnosis, prescription code, etc.

Each app was assessed by 3 persons [AR, KE, SW] using the long questionnaire (see section results), whereby the persons cross-checked their results within each category of Apps.

## Results

### Overview

From the comprehensive evaluation criteria, we derived 2 questionnaires (short and long) to enable an informed and self-determined decision regarding the assessment of these apps. All questions in the following tables are to be answered with (yes/no) along with a detailed description. The responses serve as a guidance for the individual decision-making process regarding the app’s utility and quality.

### Short Questionnaire

The short questionnaire summarizes the questions, which were most often answered with “no” in the evaluation of the long questionnaire and were rated as very important in the focus group and survey mentioned above. Table 1 shows the 6-items short questionnaire with an explanation of the reason for the importance as well as meaningful advice on how to self-assess the questions quickly.

**Table 1.** Short questionnaire with the most relevant questions for informed decision-making for health apps.

Category	Answer	Why is this important?	Meaningful advice
Imprint:Does the App have an imprint indicating the App’s manufacturer?	yes or no	In Austria, there are legal requirements regarding the “Imprint” that must be complied with.	Often you can find the imprint in the settings or in the Terms and Conditions (Terms and conditions) of the app. WARNING: Many apps do not have an imprint. This could be an indication that the app is not trustworthy. An imprint must contain the following information: Name or company according to the commercial register, Address, Contact information (eg, email address or phone number).
Imprint:Is the contact information in the imprint complete?	yes or no		
User account: Do I need to create an account to use the App	yes or noAttention: Both can have advantages and disadvantages!	If you have to log in to an app, the provider can more easily associate the data directly with you. This is especially true if you need to provide additional personal information (name, address, phone number, etc.) during registration. If you don’t need to create a user account, that can also be an advantage. You can use the app anonymously. Warning: If you delete an app without a user account or use another device, your data may be lost.	If you need to create a user account, the manufacturer can store your data and uniquely identify you. If you need to log in with a password, no one else can use the app under your name. Hint: Always use a secure password for health apps.
User account: Can you easily delete this account again?	yes or no		The function to delete the account is often difficult to find. TIP: Check the app’s settings. Additional steps may be required to delete the user account, such as entering the password.
Data Privacy:Is it specified which specific data the App collects? (for example: Name, Location, etc.)	yes or no	There are legal requirements for data protection in Austria. These requirements must also be complied with in apps.	You usually find information about data privacy in the privacy policy and/or the Terms and Conditions (AGB). Sometimes you may not find this information directly in the app but during the registration process. The privacy policy must specify the categories of data processed (eg, health data). WARNING: Often, data not related to the app’s purpose is processed (eg, GPS data in a flashlight app).
Data Privacy:Is it indicated whether this data is shared with third parties?	yes or no	It is best if an app does not share your data with third parties. If data is shared, it must be indicated.	Sharing of Data with Third Parties: App providers often take the liberty of sharing data with third parties if it is convenient for them. Tip: Searching for the word “Third parties” will direct you to the relevant section. If data are shared with third parties, it must be stated to whom the data is transmitted. Data are often stored outside the EU and are therefore subject to different data protection laws. If you search for the word “USA,” you can find out if your data isarestored in the United States. Different data protection laws Apply there.
Labeling of Advertising:Are paid offers in the App labeled as “Advertisement” or “Ad”?	yes or no	Advertising must be labeled and clearly recognizable as such.	To access all contents of the app, a paid subscription may be necessary. Paid subscriptions in apps are often not labeled as advertisements. Subscription offers can be annoying and are often difficult to dismiss. Paid subscriptions are often automatically renewed. Warning: Renewal is often agreed upon when subscribing. Always read carefully. When comparing the costs of different apps, always consider the total annual costs without initial discounts. Tip: Pay attention to any cancellation deadlines and minimum terms. Subscriptions can be canceled either directly in the app or on the providers’ websites.
App Function:Is the App’s function adequately described?	yes or no	The function of an app is often the decisive reason for using it. Therefore, the app’s function should align with your personal goal.	You can usually read the app’s description before downloading. Warning: Goals and target groups of the app are often not specified. Pay attention to advertising promises in the description. Promises are often not fully realized. Tip: Check if the app’s function is usable in the country where you reside. For example, apps in the “Nutrition” category may use databases from the USA, which may not be usable in Austria. Often, a supposedly German-language app also displays content in English.
App Function:Does the description match reality?	yes or no		
Information:Is the information contained in the App scientifically supported?	yes or no	Health information should meet certain quality criteria. In any case, scientific evidence (sources) should be provided for claims.	Pay attention to source references for information in the app. Warning: Often, no sources are provided for health information, recommendations, or claims. This means the information could be fabricated. Pay attention to the timeliness of health information in apps. Information is often outdated and no longer in line with the state of science (eg, in nutrition apps). Often sources are provided that don‘t directly match the app‘s function.

### App Selection

The selected apps to be tested against the long questionnaire is shown in Table 2. Detailed evaluation is not to be published due to possible violation of commercial law.

**Table 2.** Health apps that were selected for evaluating the questionnaire.

Category and name of the app	Manufacturer
Nutrition	
Kalorien, Fett & Eiweißzähler	Virtuagym
Lifesum: Gesunde Ernährung	Lifesum
feastr Ernährungsplan Abnehmen	feastr
MyFitnessPal: Kalorien Tracker	MyFitnessPal Inc.
Yazio: Kalorien Zähler & Diät	Yazio
Kalorienzähler von FatSecret	FatSecret
Was ich esse	Bundesanstalt für Landwirtschaft und Ernährung
MySwissFoodPyramid	BA für Lebensmittelsicherheit und Veterinärwesen
Nutrilio: Gesunde Ernährung	Habitics
Foodiary: Rezepte und Diät	Foodiary
Movement	
Dehnung und Flexibilität	Leap Fitness Group
StepsApp Schrittzähler	Stepp-App
7-minute workout	Perigee
Nike Run Club: Laufcoach	Nike, Inc.
Schrittzähler - Health, iStep	Leap Fitness Group
Google Fit: Aktivitätstracker	Google LLC
Nike Training Club: Übungen	Nike, Inc.
Adidas Running: Lauf-App	Adidas Runtastic
SPAR Health Coach	Spar Österreich
Activity Tracker Schrittzähler	Bits & Coffee
Mental health	
Breeze: Mental Health	Basenji Apps
Voidpet Garden: Mental Health	Voidpet
VOS: Wellbeing Plan & Tagebuch	VOS.health
Finch: Self Care Pet	Finch Care Public Benefit Corporation
Mindshine: Mental Health Coach	Mindshine (Greator GmbH)
I am - affirmaties	Monkey Taps LLC
Amaha: anxiety self-care	Amaha Health
Wysa: Anxiety, therapy chatbot	Touchkin
BetterMe: Seelische Gesundheit	BetterMe Limited
Headspace: Medidation & Schlaf	Headspace for Meditation, Mindfulness and Sleep
Symptom tracker	
Ada - Check Deine Gesundheit	Ada Health
Flo Perioden- & Zyklus-Kalender	Flo Health Inc
MyTherapy Tabletten Erinnerung	MyTherapy
Chronic Insights	Chronic Insights Ltd.
Symptom Tracker	CareClinic Tracker & Reminder
Symptom Checker & Medication - FriendsApp Listing	FriendsApp Listing
mySymptoms Food Diary	SkyGazer Labs Ltd
Migraine Buddy	Healint
Symptomate	Infermedica
Bearable Symptom & Mood Tracker	Bearable Ltd

### Evaluation Results

This chapter summarizes the evaluation results of the long questionnaire against the selected apps (Table 3).

**Table 3.** Evaluation results summarized.

Question items	Nutrition	Exercise	Mental health	Symptom checker
Legally required imprint available	4 out of 10 apps	4 out of 10 apps	1 out of 10 apps	5 out of 10 apps
Privacy policies that allow for the disclosure of health data to third parties [24]	All international operating apps	All international operating apps Privacy policies are sometimes unclear and contradict the information provided in the app stores by the manufacturers	All international operating apps Consent to the terms of use is implicitly obtained for most apps by using the app.	User consent is mostly requested via checkboxes.
Addressing the sensitivity of health data	is not explicitly addressed	is not explicitly addressed	is not explicitly addressed	It is recommended that the app does not substitute the doctor
App tracking	All apps	All apps	All apps	not available
Target group definitions	Usually not available	Usually not available	Not available	One app only mentions in the disclaimer that it is intended for users in the United States.
Other	German apps have good privacy policies and refer to scientific sources For many apps, a newsletter was simultaneously subscribed to without explicit consent	All apps reserve the right to collect at least pseudonymized user data Making unsubstantiated claims (“Yoga for Weight Loss”, “Correction of Bow Legs”, “Relieving Menstrual Cramps through Breathing”, ...)	Two out of 10 apps refer to scientific backgrounds for their implementation Many of the apps aim to subscribe to a subscription after or during the registration process, which usually ends in a costly annual subscription of 20‐130 Euros per year (~20$-130 US$ per year) after a certain free trial period. Since most apps are financed through subscriptions, many do not use advertising placements.	Some apps allow for the export of data, which can be presented to the doctor as a report in PDF or CSV format or, in very few cases, directly provided to the doctor via a link to the health data. Many apps are free. Subscriptions usually cost between 7 and 40 EUR per month. When entering symptoms, some apps provide a wide range of possible causes. This could lead to overdiagnosis and unnecessary interventions.

### User Survey

A quantitative survey with 414 participants was conducted to evaluate the value of health app features for achieving different goals from users’ perspectives, aiming for a balanced gender identity, age, and prior experience with health apps. The analysis focused on the relevance of goals such as support for physical activity (fitness), weight management, nutrition, mental health, and the monitoring of physical and mental symptoms. The findings showed that health apps in the “fitness/activity” and “symptom checker” categories were considered most important, while mental health apps were rated as less important. The ratings were influenced by gender, age, and the presence of chronic conditions, and prior experience with health apps also played a role. Women rated health app functions as more important than men, with a focus on supporting physical health and achieving weight management goals. Men prioritized physical activity support, while younger participants rated the importance of health app features higher than older ones, particularly for fitness-related goals. Individuals with chronic conditions placed more emphasis on functions promoting physical health and were more likely to pay for health apps.

## Discussion

### Principal Findings

Despite efforts in initiatives like Label2Enable [10], “So far, no unified concept has emerged to provide users with orientation regarding the quality of health apps. Different approaches with varying underlying criteria, often pursued by commercial entities, are being followed (eg, evaluation instruments for users, certifications, quality seals, test reports)” [12]

Our aim with this work has been to provide users with a unified concept for orientation regarding the quality of health apps, knowing well that the comprehensive instruments offered by Health technology assessments are not usable for users. The goal of this work was to create a questionnaire that could be used to evaluate 10 apps each in the areas of nutrition, exercise, mental health, and symptom checkers. The key points were summarized in the short questionnaire and supplemented with helpful tips and a “Meaningful advice” column, enabling users to assess the quality of an app in a short amount of time.

The user survey revealed that data privacy and security considerations played a minor role for most respondents, contrary to the questions outlined in the ”Short Questionnaire .” This could be due, on one hand, to the general perception of loss of control over one’s data, and on the other hand, the impacts of data privacy and security are not directly and immediately discernible to users. Data leaks with potentially negative consequences for individuals may only become known after a considerable period of time, and their effects may unfold gradually.

Additional education and information on “digital self-determination or self-defense” could help address this issue, enabling users to make clear and informed decisions about whether they prefer data-minimizing apps over those whose primary goal is to collect and market metadata. Simple questionnaires, as presented in this work, can raise awareness regarding data privacy and privacy, especially concerning sensitive health data, which may be perceived as critical in connection with initiatives like Elektronische Gesundheitsakte (ELGA) [25] but less so in the context of apps.

Digital health literacy already plays a role in selecting a suitable digital health applications, not only for users but also for therapists and medical professionals. Choosing a suitable, safe, and effective application from the multitude of digital health apps is a significant challenge. Currently, many users (35% in the user survey) select digital health applications based solely on star ratings in app stores, which have limited reliability [26]. In this context, the use of a questionnaire makes sense, especially regarding expected features and their evidence-based justification.

Apps are often downloaded based on advertising on social media, app stores, and recommendations from family and friends. This indicates that a quality-assured app recommended by a doctor could have significant potential for user adoption. Therefore, it is essential that reviews of these apps are quality-assured and reliable. This applies to both health apps and medical apps, which may target vulnerable groups such as people with chronic illnesses. Considerations regarding the German DiGAs [27] were taken into account throughout the project. Still, the outcome of this work is more geared towards private users rather than representatives of the health system. Nonetheless, we are pleased to note that Digitale Gesundheitsanwendungen (DiGAs - in English: digital health applications) have found a place in the preliminary version of the Austrian eHealth Strategy “M1.7 Creation of a unified process for the evaluation of Digital Health Applications and subsequently their introduction into standard care when demonstrably beneficial” [28]. This also applies to statements from the professional assessment of health apps. “The information offered is too extensive for laypeople”—here, too, a standardized evaluation and recommendation by authorities and health care providers would be advantageous.

In general, it can be noted that Austrian app developers adhere more frequently to regulatory requirements than international developers, since they most often met the evaluation criteria, especially in terms of data privacy and imprint. In this context, we would like to appeal to the responsibility of app providers to make a positive contribution to societal challenges A white paper in line with “Good App Development Practice” or regional requirements from pp stores could introduce even quality-assurance restrictions, particularly regarding regulatory frameworks (eg, explicitly demanding a legally compliant imprint) as collected in the results of this work.

### Limitations

The selection of apps to be evaluated based on the established and prioritized criteria proved to be more challenging than initially anticipated. This was partly due to the lack of qualified sources for the previously desired criterion of “most commonly used apps in Austria,” requiring us to resort to an exploratory approach. Furthermore, the apps found as a result could not be fully tested and evaluated by all participants, as even during the implementation period, some apps were no longer available in the app stores for specific operating system versions. Nevertheless, a common denominator for joint evaluation had to be found.

In general, we want to emphasize that a framework for evaluating apps was established and applied, aiming to empower users to independently assess and evaluate both current and new apps.

We deliberately refrained from presenting individual apps or manufacturers in a positive or negative light.

Furthermore, we want to emphasize that our study has not been aimed at replacing the traditional instruments of Health Technology Assessment, as the evaluation criteria were created for the purpose mentioned above. However, this evaluation did not involve searching for clinical studies to demonstrate the effectiveness of the apps, and the apps were not assigned to risk classes according to Medical Device Regulation [29]. As such, for a comprehensive assessment of a health app, fulfilling the requirements of evidence-based medicine, further important domains such as clinical effectiveness, clinical safety, costs, organizational and ethical aspects need to be assessed.

### Conclusions

In a rapidly changing landscape such as the app market, it is therefore even more important to have a solid, easy-to-use tool for evaluating health and medical apps at hand. We are aware that this tool may not be sufficient for evaluation by authorities; however, we would like to note that it can provide a good basis for it. We would like to support further research and projects in this direction.

## Abbreviations

DiGA: Digitale Gesundheitsanwendungen
ELGA: Elektronische Gesundheitsakte
HSC: health system challenges
MARS: Mobile App Rating Scale
mERA: mobile health (mHealth) evidence reporting and assessment
mHealth: mobile health
WHO: World Health Organization
